# The Impact of Anesthetic State on Spike-Sorting Success in the Cortex: A Comparison of Ketamine and Urethane Anesthesia

**DOI:** 10.3389/fncir.2017.00095

**Published:** 2017-11-29

**Authors:** K. Jannis Hildebrandt, Maneesh Sahani, Jennifer F. Linden

**Affiliations:** ^1^Cluster of Excellence Hearing4all, University of Oldenburg, Oldenburg, Germany; ^2^Department of Neuroscience, University of Oldenburg, Oldenburg, Germany; ^3^Gatsby Computational Neuroscience Unit, University College London, London, United Kingdom; ^4^Ear Institute, University College London, London, United Kingdom; ^5^Department of Neuroscience, Physiology and Pharmacology, University College London, London, United Kingdom

**Keywords:** spike sorting, anesthesia, population dynamics, coherence analysis, auditory cortex

## Abstract

Spike sorting is an essential first step in most analyses of extracellular *in vivo* electrophysiological recordings. Here we show that spike-sorting success depends critically on characteristics of coordinated population activity that can differ between anesthetic states. In tetrode recordings from mouse auditory cortex, spike sorting was significantly less successful under ketamine/medetomidine (ket/med) than urethane anesthesia. Surprisingly, this difficulty with sorting under ket/med anesthesia did not appear to result from either greater millisecond-scale burstiness of neural activity or increased coordination of activity among neighboring neurons. Rather, the key factor affecting sorting success appeared to be the amount of coordinated population activity at long time intervals and across large cortical distances. We propose that spike-sorting success is directly dependent on overall coordination of activity, and is most disrupted by large-scale fluctuations in cortical population activity. Reliability of single-unit recording may therefore differ not only between urethane-anesthetized and ket/med-anesthetized states as demonstrated here, but also between synchronized and desynchronized states, asleep and awake states, or inattentive and attentive states in unanesthetized animals.

## Introduction

Extracellular *in vivo* recordings from many cortical areas in rodents have produced abundant evidence for changes in patterns of single-unit activity under different anesthetics (Zurita et al., 1994; Kisley and Gerstein, 1999; Gaese and Ostwald, 2001; Cotillon-Williams and Edeline, 2003; Murakami et al., 2005; Potez and Larkum, 2008; Huetz et al., 2009; Marguet and Harris, 2011; Sellers et al., 2015; Lissek et al., 2016), or in different behavioral or sleep states (Manunta and Edeline, 1999; Otazu et al., 2009; Harris and Thiele, 2011; Schneider et al., 2014; McGinley et al., 2015; Vinck et al., 2015). However, to the best of our knowledge, the influence of changes in brain state on the success of efforts to isolate single-unit spikes from extracellular recordings (“spike sorting”) has not been systematically explored or quantified.

Spike sorting is greatly complicated if recorded spikes overlap in time or are masked by coherent noise, because these distortions disguise otherwise unit-specific spike shapes on which spike sorting is based (Sahani, 1999; Wehr et al., 1999; Harris et al., 2000). Since anesthetic state alters cortical dynamics (Cotillon-Williams and Edeline, 2003; Britvina and Eggermont, 2008; Curto et al., 2009) and presumably synchrony of cortical activity (Luczak et al., 2007; Pachitariu et al., 2015), we wondered whether anesthetic agents might differ in their impact on the sortability of extracellularly recorded data. If so, then attempts to define how single-unit responses to sensory stimuli are affected by changes in alertness, brain state, or indeed anesthetic regime would need to take into account the influence of those changes on the accuracy of single-unit recording in the first place.

We studied sortability of spike data in the auditory cortex, where the problem of overlapping activity is particularly acute since neural firing is highly coordinated (Loebel et al., 2007; Harris et al., 2011) and precise in time (Wehr and Zador, 2003). We compared spike sorting of neural activity in the auditory cortex under two anesthetics that are commonly used in studies of the rodent auditory system, and which are known to induce different patterns of gross population activity (Luczak et al., 2007; Rennaker et al., 2007): ketamine (in combination with the additional anesthetic and analgesic medetomidine) and urethane.

Our results indicate that cortical brain state can have a major impact on spike-sorting success. Moreover, we find that a primary cause for deterioration in spike-sorting success is not changes in inter-event intervals (IEIs) statistics (such as increased overlaps or burstiness), but increased coordination of neural activity at longer spatial and temporal scales.

## Materials and Methods

All experiments were performed under a license approved by the UK Home Office in accordance with the United Kingdom Animal (Scientific Procedures) Act of 1986. Fourteen male CBA/Ca mice between 8 and 21 weeks of age were used in these experiments. Extracellular electrophysiological recordings were obtained from the right auditory cortex using multi-electrode probes inserted at a total of 52 sites in eight mice anesthetized with ketamine/medetomidine (ket/med) and 27 sites in six mice anesthetized with urethane.

### Surgical Procedure

For ket/med anesthesia, an initial dose of 0.04 ml per g body mass of a mixture of ketamine (10 mg/ml) and medetomidine (0.083 mg/ml) was administered by intraperitoneal injection. Anesthetic state was maintained at a stable level by continuous intraperitoneal infusion of a ketamine/medetomidine mixture (10 mg/ml ketamine, 0.042 mg/ml) at a flow rate of 0.005 ml/(g*h). Anesthesia levels were monitored using whisker twitch and pedal withdrawal reflexes assessed approximately every 30–60 min.

Urethane anesthesia was initiated with an intraperitoneal dose of 1.9 g/kg body mass using a 20% w/v urethane solution (i.e., 1 g urethane per 5 ml sterile water). Anesthesia levels were monitored using whisker twitch and pedal withdrawal reflexes, and anesthesia was supplemented by a dose of 950 mg/kg body mass if needed. Supplementary doses of urethane were usually necessary only when the experiment lasted longer than 4–5 h. In experiments conducted under urethane, a tracheotomy was performed to prevent breathing problems that can occur in urethane-anesthetized mice.

In addition to the anesthetic agent, dexamethasone (10 mg/kg body weight) and atropine (0.05 mg/kg body weight) were administered subcutaneously to reduce brain edema and bronchial secretions, respectively. Ringer’s solution was administered subcutaneously throughout the experiment (0.1 ml every 1–2 h) to maintain hydration. Breathing and temperature were monitored, and a homeothermic blanket system (Harvard Apparatus) was used to maintain body temperature at 37.5 ± 0.5°C.

Once anesthetized, mice were placed in a nose clamp to immobilize the head and rotated at an angle of ~45° onto the left side so that the right auditory cortex was facing sideways and upward. The scalp was transected to expose the skull, and a craniotomy was made over the right temporal lobe by removing the skull bounded by the temporal ridge, the lambdoid suture and the ventral and rostral squamosal suture. Ringer’s solution was placed over the craniotomy to keep the exposed cortex moist, and multielectrode penetrations were made through the dura.

### Recording Procedure

Extracellular recordings were collected using a silicon multi-electrode with 16 sites, organized in tetrodes along two shanks, containing two tetrodes each. Both between-shank and within-shank tetrode distances were 150 μm. Electrodes within a tetrode were organized in a diamond shape with spacing of 25 μm between adjacent sites (A2x2-tet-3 mm-150-150-121-A16, NeuroNexus Technologies).

The multi-electrode was inserted orthogonal to the cortical surface and advanced until tetrodes were positioned 350 and 500 μm below the surface. All recordings were performed with the multi-electrode in a consistent orientation, with the two shanks positioned along the rostral-caudal axis of A1. The multi-electrode signals were amplified and digitized (Medusa RA16SD, RX-5, Tucker-Davis Technologies), sampling at 25 kHz with a high-pass filter cutoff at 600 Hz, and collected using OpenEx (Tucker-Davis Technologies). Local field potential (LFP) was not recorded for reasons unrelated to the purpose of the present study; therefore we were not able to analyze synchronized and desynchronized states in these recordings. The location of multi-electrode penetrations within the fields of auditory cortex was determined on the basis of tonotopy and response properties (Stiebler et al., 1997; Linden et al., 2003; Guo et al., 2012; Kanold et al., 2014), targeting A1.

### Acoustic Stimulation

Experiments were conducted in an acoustic isolation booth (Industrial Acoustics Company). All stimuli were presented free-field (FF1 speakers, Tucker-Davis Technologies) to the ear contralateral to the recording site, with a sound-attenuating plug inserted into the ipsilateral ear. Frequency response areas for each recording site were estimated using 100 ms tones ranging from 4 kHz to 80 kHz in frequency, 8 tones per octave and from 10 dB to 80 dB SPL in level in 10 dB steps. All stimuli were synthesized using a digital signal processing unit with a 195,312.5 Hz sampling rate (RX-6), attenuated if necessary (PA-5) and then passed through a stereo amplifier (SA-1; all Tucker-Davis Technologies). Stimuli were designed and controlled using a combination of Matlab (The MathWorks) and OpenEx (Tucker-Davis Technologies).

### Data Analysis

Statistical tests were performed two-sided unless otherwise specified, and results were described as significant for *p* < 0.01.

Action potentials were classified using a latent-variable spike-sorting algorithm (Sahani, 1999; Wehr et al., 1999). Sorting was based on spike snippets from all four sites of each tetrode. For the analysis of multi- vs. single-site sorting, a single site from each tetrode was drawn for each recording and spike sorting was based on the snippets recorded at that site. Multi-unit clusters as reported here were derived by thresholding only, and ignoring the categorization provided by the spike-sorter. Single-unit classifications were accepted only if the spike-sorter reported both false-negative and false-positive rates <5%. In addition, clusters were rejected if the proportion of IEI shorter than 1.5 ms was >1% of the entire distribution.

#### Statistics of Event Trains

In order to quantify the burst-like nature of the events extracted by thresholding, we used the C_V2_ (Holt et al., 1996):

(1) $$C_{V2}=\frac{1}{n-2}\sum_{i=1}^{n-2}2\frac{\left|\Delta t_{i+1}-\Delta t_{i}\right|}{\Delta t_{i+1}+\Delta t_{i}},$$

where *Δt*_i_ is the IEI between event times *t*_i_ and *t*_i+1_ and *n* the number of events. The C_V2_ is 0 when all IEI are uniform in duration, and increases toward 2 when successive IEI differ in duration; therefore this measure provides a simple index of “burstiness”.

#### Variance of Voltage Traces Around Threshold Events

In order to quantify the variance around unsortable multi-unit events, all threshold crossings that could not be assigned to a single-unit cluster were collected for each tetrode and penetration. Variance of the voltage traces was computed for each point in a time window centered on these threshold crossings. Variances were then normalized to the mean variance surrounding random time points during the recording; i.e., we normalized the variances around threshold-crossing events by the mean variance around a similar number of randomly chosen time points. Variances around spike events from well-separated single units were computed in the same way, but relative to the event times for each sorted unit rather than to threshold crossings.

#### Quantification of Coordination of Activity

Coordination of neural activity was quantified as the coherence between the raw voltage traces at different sites of the multi-electrode probes. We used Hamming windows of length 1024 samples (~24 ms), overlapping by 50%, to compare the frequency content of the signals between the sites:

(2) $$C_{xy}(f)=\frac{|P_{xy}(f){|}^{2}}{P_{xx}(f)P_{yy}(f)}.$$

where *C*_xy_ is the the average coherence between sites *x* and y, *P*_xy_ the cross-spectral density and *P*_xx_ and *P*_yy_ the autospectral densities of the signals at each site. To quantify within-tetrode coherence, we averaged the coherences computed using all six possible combinations of electrode sites within a tetrode. To obtain a measure of between-tetrode coherence based on the same number of electrode site comparisons, we randomly drew one electrode site from each tetrode of the pair and computed the coherence, then repeated this process six times and averaged the results. Data presented here are mean coherences across all experiments.

To analyze the cross-correlation of threshold crossings between tetrodes, crossing times at each tetrode were convolved with a Gaussian with 2 ms standard deviation. The resulting traces were normalized by dividing them by the mean rate of the threshold crossings for the tetrode. The cross-correlation between these normalized traces was then computed for different tetrode combinations. For evaluation of spontaneous activity, only threshold crossings in a 50 ms window preceding an acoustic stimulus by at least 200 ms were used. For evoked activity, only crossings in the first 50 ms after stimulus onset were analyzed.

### Generation and Evaluation of Synthetic Data

Synthetic data for the simulation of the effect of coherence and variance was synthesized as follows. Background activity for both high global coherence and control (low coherence) was simulated by summing extracellular spikes taken from a pool of 100,000 successfully sorted spikes from a large set of extracellular recordings. At each time step, the number of spiking units was either constant (control) or modulated by uniformly distributed noise (high coherence). The number of contributing units per time step was matched between the conditions and adjusted to a mean spike rate of 10 Hz per unit. Modulation depth of the high-coherence situation was adjusted to match coherence for the ketamine data. In order to keep local coherence at the same level for both conditions, spike subsets contributing to the background for the different sites of the tetrode partially overlapped (25% overlap). This was not the case for the high-coherence data. Foreground spikes were taken from successfully sorted single-unit recordings that were not part of the background pool.

The simulation was run in three versions: (1) low signal-to-noise ratio (SNR, i.e., foreground spikes to background) with background variance and spike amplitudes matching the average ketamine data; (2) high SNR with spike amplitudes twice as large as in the low SNR condition; and (3) a version where high- and low-coherence backgrounds were scaled to have the same variance relative to spike amplitudes in order to test the contribution of higher variance and the temporal structure of the background.

Subsequently, spike sorting was run using the same routines as for the original data. Spike-sorting success was quantified as the percentage of originally entered foreground spikes correctly assigned to the clusters.

## Results

### Spike Sorting Is More Successful Under Urethane than Ket/Med Anesthesia

We compared spike sorting under two anesthetics commonly used for the study of the rodent auditory system: ketamine (in combination with the additional anesthetic and analgesic medetomidine) and urethane.

**Figure 1:**
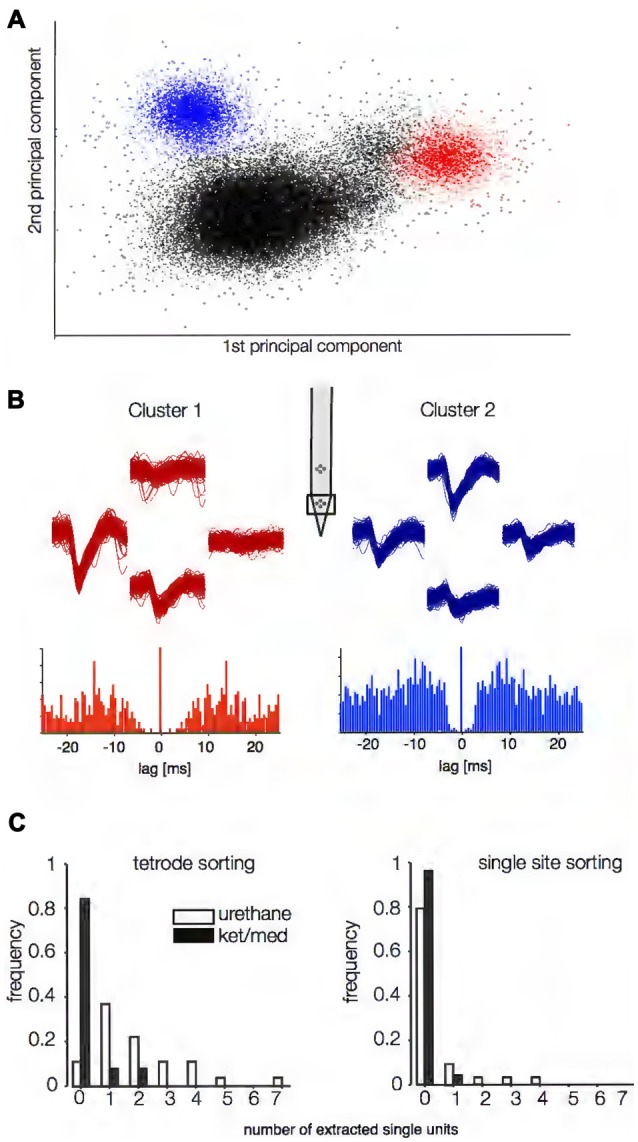
Spike-sorting success under two different anesthetics. Putative single units were judged to be successfully extracted using criteria based on separability of waveforms in principal component space **(A)** and the shape of the inter-spike interval (ISI) autocorrelation function **(B)**. In **(A)** the first two principal components of the spike snippets are plotted for an example recording obtained under urethane anesthesia. In this example, two single units could be successfully extracted (blue and red dots, respectively). In **(B)**, the autocorrelation functions for the ISIs for the two units are displayed. The traces above the autocorrelation panels show voltage traces at the different sites of the tetrodes (inset) for both units. **(C)** Spike-sorting success for all recording sites obtained under ketamine/medetomidine (ket/med, *n* = 52) and urethane (*n* = 27) anesthesia. The left panels depict sorting success if all four sites of the tetrode (**B**, upper panels) were used (*p* = 2.8*10^−10^, Wilcoxon rank-sum test). For the right panel, the same data was analyzed, but using only one site per tetrode for spike sorting (*p* = 0.0094, Wilcoxon rank-sum test).

Data were obtained from anesthetized mice using silicon multi-electrodes with four tetrodes (two on each of two silicon shanks) separated by 150 μm both vertically and horizontally. The electrodes were inserted perpendicularly at various sites in the core auditory cortex, with the tetrodes located about 350 and 500 μm beneath the cortical surface. We identified successfully isolated single units in the spike-sorting output using two objective criteria. First, we evaluated the probabilistic assignment of spike waveforms to clusters (Sahani, 1999; Wehr et al., 1999) and required that both the expected false positive and false negative rates be below 5%. Second, we required that no more than 1% of the inter-spike intervals (ISIs) violate a putative refractory period of 1.5 ms. An example of successfully sorted spike data is given in Figures 1A,B. For this particular dataset, the spike-sorting algorithm originally identified 10 different clusters, eight of which did not meet the isolation criteria and were therefore rejected (black dots in Figure 1A). Distinct spike shapes at the different sites of the tetrode and autocorrelation functions of the spike times of two remaining clusters supported their single-unit origin (Figure 1B).

Using these strict criteria, our automated protocol successfully extracted at least one single unit from 88.9% (24/27) of tetrode recordings when the animal had been anesthetized with urethane, finding two or more single units 48.1% (13/27) of the time (Figure 1C, left panel). By contrast, only 15.4% (8/52) of tetrode recordings yielded any single units under ket/med anesthesia, with two units isolated from just 7.7% of recordings. We were never able to isolate more than two single units from a tetrode recording under ket/med anesthesia. Average yield was 2.05 single units per tetrode under urethane, but only 0.23 under ket/med anesthesia, an almost nine-fold difference (*p* = 2.8*10^−10^, Wilcoxon rank sum test). If spike sorting was performed based only on data from single sites of the tetrodes, sorting success under urethane anesthesia fell to 22.2% of recording sites (6/27), while sorting success under ket/med decreased further to 3.9% of recording sites (2/52; Figure 1C, right panel, *p* = 0.0094). Thus, spike sorting was much more successful under urethane than ket/med anesthesia, and critically depended on the simultaneous use of waveform data from multiple sites from the silicon multi-electrode.

The average cortical placement and electrical properties of the electrodes did not differ between experiments, making it likely that the significant differences in sorting success resulted from anesthesia-induced changes in the patterns of cortical activity, and consequent changes in the way signals from different cells combined to form the extracellular potential. We therefore sought to understand the nature of the change in cortical activity between ket/med and urethane anesthesia that could account for the difference in sorting quality.

### Neither “Burstiness” Nor Overlapping Spikes Account for Differences in Sorting Success

Previous authors have remarked on the “burstiness” of auditory cortical activity in the ket/med preparation (Eggermont and Smith, 1996), as well as the increase in population-wide synchronized-state activity induced by similar ketamine-based anesthesia combinations (Pachitariu et al., 2015). Others have observed that evoked auditory responses in both anesthetized and awake animals may be tightly coordinated within a column (Loebel et al., 2007; Harris et al., 2011) and precise in time (Wehr and Zador, 2003). Put together, these observations suggest that cortical activity in the ket/med preparation might become dominated by coordinated bursts of spikes within local groups of neurons.

One possible consequence of such local bursts might be that action potentials in the cells very near the electrode tip often occur within a few milliseconds of each other. In this case, the supra-threshold or “foreground” spike waveforms generated by these nearby cells would frequently overlap in time. A very high proportion of such foreground waveform overlaps almost always confounds spike sorting (Sahani, 1999). Even when an algorithm is designed to resolve overlapped waveforms (Gozani and Miller, 1994; Lewicki, 1998; Sahani, 1999; Ekanadham et al., 2014), practical success in identifying cell-specific waveform shapes almost always depends on having a large enough set of uncorrupted examples of each waveform in the data set (Sahani, 1999).

We used the *C*_V2_ measure of Holt et al. (1996), applied to the IEI between all supra-threshold events without sorting, to verify that foreground activity in the ket/med preparation did indeed tend to contain more spike bursts. The *C*_V2_ is designed to quantify the irregularity of IEIs while factoring out the effect of slow variations in mean firing rate; values greater than 1 indicate that more events occur in bursts separated by long inter-burst intervals than would be expected from an inhomogeneous Poisson process.

**Figure 2:**
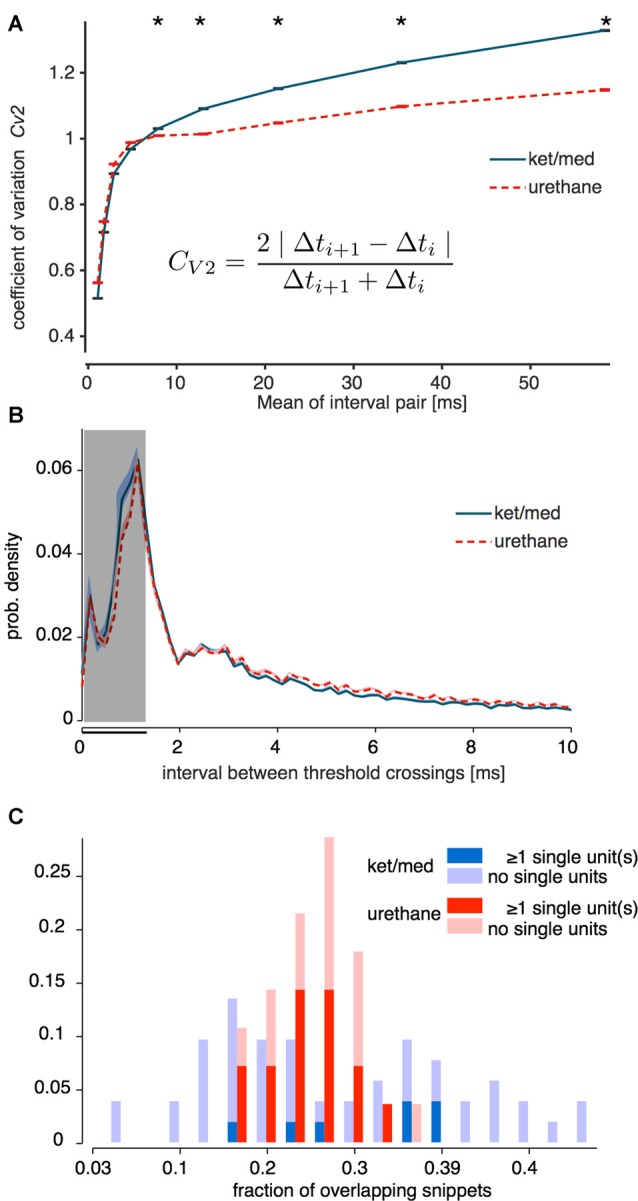
Overlap of spike snippets. In **(A)**, the C_V2_ (see inset) is plotted as measure of the irregularity (“burstiness”) of the inter-event intervals (IEIs) of the snippets used for sorting under ketamine/medetomidine (ket/med, blue line) and urethane (dotted red line) anesthesia. Error bars depict standard error of the mean (SEM) on the vertical axis; SEM is in the range of the thickness of horizontal cap lines. Asterisks mark data points for which the C_V2_ under ketamine is significantly larger than under urethane (one-sided Wilcoxon rank-sum test, *p* < 0.001). **(B)** Mean IEI distribution for all recordings of the unsorted events (blue/red shaded areas: SEM). The gray area indicates the criterion for minimal IEIs used to classify sorting of a unit as successful (Figure 1B). In **(C)** the distribution of the fraction of overlapping snippets for urethane (red) and ket/med (blue) is displayed. Successful and unsuccessful sorts are plotted separately in different dark and light shades, respectively (Kolmogorov-Smirnov test for difference in distributions: ket/med, *p* = 0.15, urethane, *p* = 0.30).

As expected, we observed a significantly higher *C*_V2_ in the ket/med preparation than under urethane for intervals >5 ms (Figure 2A; one-sided Wilcoxon rank-sum test, *p* < 0.001). However, higher *C*_V2_ under ket/med emerged in pairs of intervals with a mean of 5 ms or more (*p* > 0.5 for intervals <5 ms). Furthermore, the distribution of the IEIs themselves (rather than the serial correlation between them) differed little between the two anesthetics (Figure 2B). The proportion of IEIs shorter than the duration of spike waveform used for sorting (1.3 ms, black bar in Figure 2B) was 33% under ket/med anesthesia and 29.5% under urethane. This small difference in the overall proportions of overlapping foreground waveforms under the two anesthetics was dwarfed by the variance in that proportion across both groups of recordings (Figure 2C). Thus, if the preponderance of foreground overlaps was the primary determinant of spike-sorting success, we would expect to see a strong link between the proportion of short IEIs in a recording and the number of cells that could be isolated from it, regardless of the anesthetic used. In fact, for each anesthetic, the proportions of short IEIs followed a distribution that did not differ between the recordings from which single units were successfully extracted and those which were unsortable (Figure 2C; Kolmogorov-Smirnov test for difference in distributions: ket/med, *p* = 0.15, urethane, *p* = 0.30).

Thus, a change in the frequency of overlapping foreground spikes seemed unlikely to drive the differences in spike-sorting success observed in the two anesthetic preparations.

### Increase in Background Variance Near Foreground Events Under Ket/Med Anesthesia

Since the data indicated that the differences in spike-sorting success between anesthetics could not be explained by differences in the incidence of foreground spike overlaps, we investigated other possible explanations. In particular, we wondered if the feasibility of isolating spike waveforms from foreground cells might also be affected by changes in burstiness or synchronization across a larger population of more distant neurons. Individual spike waveforms from such distant neurons would not exceed the spike-detection threshold by themselves; however, increased coordination in the action-potential timing would lead to a summed “background” signal of higher amplitude superimposed over the foreground spikes. This background would appear as “noise” in the process of waveform discrimination, blurring the separation between clusters of waveforms from different foreground cells. Coordinated background spikes might also sum so as to generate spurious threshold-crossings in the absence of foreground spikes.

**Figure 3:**
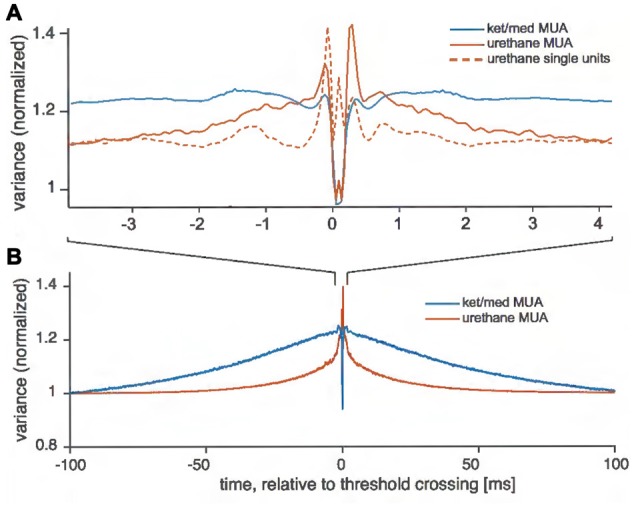
Variance of voltage around threshold crossings. Traces show the variance of the voltage around extracted events used for spike sorting at single sites of the tetrodes, normalized by the mean variance for each recording obtained at random points in time for each recording. **(A)** For ketamine/medotomidine (ket/med) anesthesia, only unsortable traces (i.e., multi-unit activity) are shown (MUA, blue solid line). For urethane anesthesia, events were split into those with successful extraction of a single unit (single units, red dotted line) and those which could not successfully be assigned to a single unit (MUA, red solid line). **(B)** Variance around threshold crossings on a longer time scale.

To investigate this possibility, we examined the variance in the electrode signal recorded near threshold crossings, separating crossings associated with spikes from isolated cells (in the urethane preparation) from those assigned to unsortable multi-unit activity (Figure 3). For successfully sorted events recorded under urethane anesthesia, the variance of the extracellular voltage signal dropped to about 110% of the long-run variance immediately preceding and following detected events (Figure 3A). By contrast, events that could not be assigned to a single-unit cluster in the urethane preparation were embedded in higher variance for an average of 3 ms both before and after the event. Under ket/med anesthesia, very few threshold crossings could be successfully assigned to a single-unit cluster (Figure 1C), and taking all threshold crossings in the ket/med preparation together, the variance was not only significantly larger than seen under urethane, but also elevated above the long-run level for a full 100 ms before and after the events (Figure 3B).

Waveforms drawn from a multi-unit cluster will almost always show larger variance than those associated with a single cells, because the variance between the different underlying waveform shapes assigned to the cluster adds to the variance of the background and any noise. However, the pattern of variance seen here, extending before and after the foreground waveforms themselves, cannot simply be explained by the amalgamation of foreground events from different cells. Instead, it is most consistent with a coordinated increase in background activity. Under ket/med, this increase in background extended over about 100 ms, consistent with the typical duration of the “up-states” of ketamine-induced synchronized activity (Pachitariu et al., 2015). For technical reasons, we were unable to directly examine the relationship between spike-sorting success and LFP characteristics in our recordings. However, our finding here that elevations in background variance around spiking events were more extended temporally under ketamine than urethane anesthesia is consistent with previous work on LFP characteristics under these two anesthetics, which have shown that cortical activity is more often synchronized under ketamine than urethane anesthesia (Luczak et al., 2007; Sakata and Harris, 2012; Pachitariu et al., 2015).

### Spectral and Spatial Statistics of Electrode Signal Variance Under Ket/Med Point to Large-Scale Coordination of Population Activity

**Figure 4:**
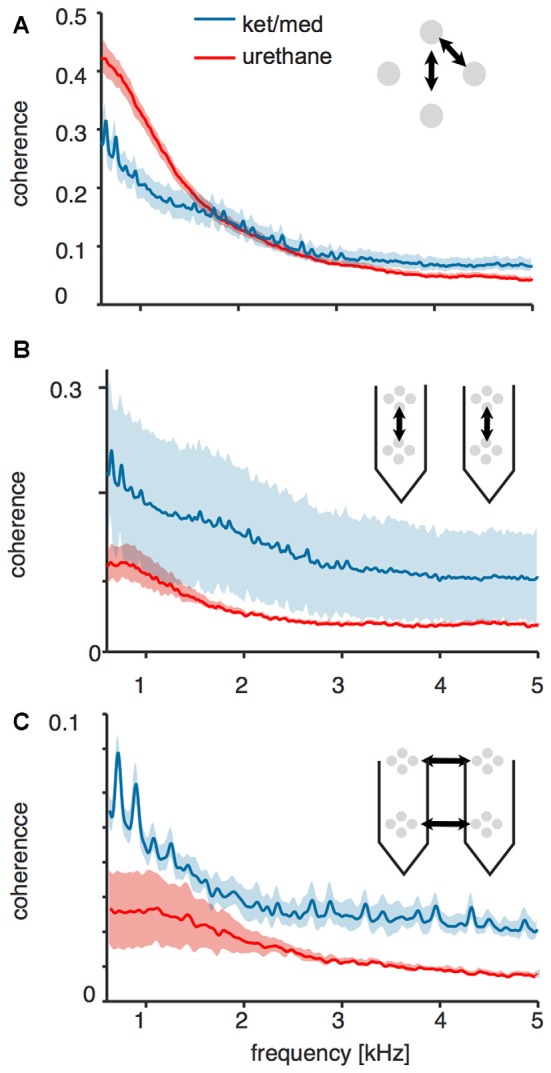
Coherence of raw voltage traces across different sites of the probes. All panels show mean ± SEM coherence of voltages across different geometries of electrode site pairs for recordings obtained under ketamine/medetomidine (blue) or urethane (red) anesthesia. **(A)** Local electrode sites within the same tetrode. **(B)** Distant electrode sites across tetrodes along the same shank of the recording probe. **(C)** Distant electrode sites across tetrodes on different shanks.

We wondered if the pattern of spatial coherence in the voltage signal measured under ket/med anesthesia was consistent with an increase in coordination of spiking within a relatively extended population of neurons. We first quantified spatial coherence over small scales and local populations by analyzing coherence of voltage signals recorded at pairs of electrode surfaces within the same tetrode. Coherence up to a frequency of about 1.5 kHz between signals recorded at electrodes within the same tetrode was lower in the ket/med preparation than under urethane (Figure 4A). Thus, the major source of the added variance (Figure 3) in ket/med recordings was not strongly correlated over a spatial scale of 25 μm, and dominated by lower frequencies. This finding suggests that the added variance around threshold crossings is unlikely to arise from nearby foreground cells, currents from which would impinge all electrodes within the tetrode, and for which higher frequencies would likely be preserved. Instead, the result suggests that increased variance around threshold crossings under ket/med anesthesia originates in cells at some distance from the electrode, with each electrode coupling to a somewhat different group.

Consistent with this suggestion of more extended spatial coordination in the ket/med preparation, the coherence between signals from these recordings measured at pairs of electrodes separated by about 120 μm vertically was almost as strong as that in electrodes only 25 μm apart, whereas under urethane anesthesia signals at this separation were much less coherent (Figure 4B). Coherence between widely spaced electrodes in different cortical columns was substantially less than within columns in both preparations, but still remained much higher under ket/med than under urethane (Figure 4C). This preservation of coherence over long distances in the ket/med preparation is unlikely to be mediated by the direct spread of electrical signals over this range, suggesting instead coordination of population activity.

**Figure 5:**
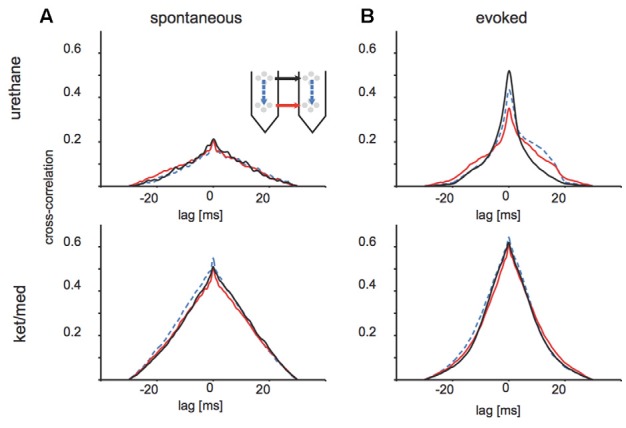
Cross-correlation of multi-unit activity across different sites of the probes. **(A)** Cross-correlations of spontaneously appearing events (multi-unit activity) under urethane (upper panel) and ketamine/medetomidine (lower panel) anesthesia. Line styles indicate geometry of the depicted pairwise comparision. Black: across shanks, superficial tetrodes; red: across shanks, deeper tetrodes; blue dotted: within shank, across tetrodes. **(B)** Same quantification as **(A)** but for evoked activity within a 50 ms window of sound onset.

Indeed, unsorted threshold crossing times at different tetrodes were more strongly correlated in the ket/med than urethane preparation, particularly in spontaneous activity (Figure 5). For spontaneously occurring threshold-crossing events, we observed almost three-fold higher cross-correlation between distant electrode sites under ket/med than urethane anesthesia, regardless of the relative positions of the tetrode pair considered (Figure 5A). Interestingly, under urethane anesthesia, cross-correlation values for activity evoked by auditory stimuli were not only higher than for spontaneous activity but also asymmetric for within-shank and deep-layer site pairings (Figure 5B, upper panel), suggesting directional spread of evoked activity along the tonotopic axis of auditory cortex. In contrast, cross-correlation values were very similar for stimulus-evoked and spontaneous activity under ket/med anesthesia (Figures 5A,B, lower panels).

### Simulation of Variance and Coherence in Synthetic Recordings Confirm Their Impact on Spike-Sorting Success

**Figure 6:**
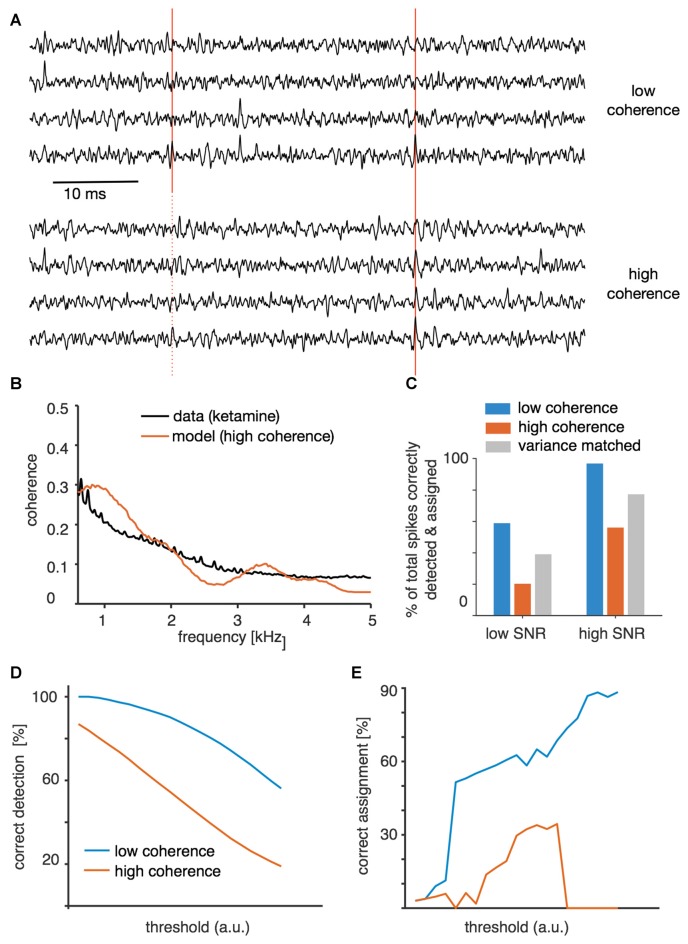
Spike sorting of synthetic data. **(A)** Example traces from the low signal-to-noise ratio (SNR) condition for high and low global coherence backgrounds, including one foreground spike correctly identified in both backgrounds (solid red lines) and one spike that was detected in the control, low-coherence background but missed in the high-coherence background (dotted red line). **(B)** Modulation of background activity was adjusted to match local coherence observed in the real data obtained under ketamine anesthesia. **(C)** Spike-sorting success at low and high SNR (mean spike amplitude relative to background variance). The proportion indicated is the overall success rate for correctly assigning the originally entered foreground spikes, and is thus a result of both detection and correct clustering. For each spike-to-background SNR level, synthetic data were generated with either low coherence, high coherence, or high coherence but with variance matched to the low-coherence case. **(D)** Success of event detection for different threshold values for the low SNR case for both high- and low-coherence backgrounds. **(E)** Success of correctly assigning successfully detected events.

In order to provide a further test of the hypothesis that higher variance around events (Figure 3) and long-range coordination (Figure 4) impede successful spike sorting, we performed a simulation in which we could directly manipulate variance and coherence of the voltage (Figure 6). Long-range coordination was simulated by temporally modulating the spiking probability of many “background” neurons, each of which had only a small impact on the background voltage by itself. The coherence that resulted from this simulation matched the profile observed under ketamine (Figure 6B). In the synthesized data set we were able to separately manipulate variance of the background and long-range coherence. Subsequent spike sorting revealed that high coherence indeed impedes spike sorting (Figure 6A). As in the real data, higher coherence was accompanied by larger variance. However, when we synthesized “variance matched” data with high-coherence background but variance matched to the low-coherence case, spike sorting improved but still did not reach the level observed in the low-coherence situation (Figure 6C). In fact, the simulation indicated that only about half of the detrimental effect of higher coordination of activity is due to the higher variance of the recorded voltage around threshold-crossing events, and the remaining effect is due to the altered temporal structure of the background. Both spike detection (Figure 6D) and subsequent classification (Figure 6E) were impaired in the high-coherence condition.

## Discussion

The identification of single units in extracellular recordings is a critical first step in many studies of sensory processing (Einevoll et al., 2012; Rey et al., 2015). Here, we investigated how brain state affects spike-sorting success in the mouse auditory cortex, by comparing the sortability of tetrode recordings obtained under two different commonly used anesthetic agents: ketamine/medetomidine and urethane. Spike sorting was much less successful under ket/med than urethane anesthesia (Figure 1). To explain the origins of this difference in sortability, we analyzed event statistics, variance of the voltage trace around a spike, and short- and long-range coordination of neural activity. Surprisingly, we found that the large differences in sortability could not be explained by changes in the frequency of coincident firing in nearby neurons (Figure 2) or the amount of locally coherent neural activity (Figure 4A). Instead, changes in large-scale coherence of cortical activity, extending over >100 ms in time (Figure 3) and >150 μm in cortical distance (Figures 4, 5), provided the best account for the differences in spike-sorting success. These conclusions were confirmed by analysis of synthetic data in which we simulated and separately manipulated variance and coherence in extracellular recordings (Figure 6). The results suggest that sortability of extracellularly recorded action potentials is undermined in anesthetic regimes and brain states that produce coordination of neural activity over long time scales and large spatial scales.

### Dependence of Spike-Sorting Success on Neural Activity Patterns

Both manual and automated approaches to spike sorting involve three key steps: (1) filtering (and sometimes “whitening”) of the extracellularly recorded voltages to improve the signal-to-noise ratio for detecting action potentials; (2) thresholding of the filtered traces and extraction of 1–2 ms long “snippets” around threshold-crossing events to extract potential spikes; and (3) clustering of snippet waveform features such as peak height or principal component amplitude to identify likely single-unit waveforms. Spike-sorting success is often greatly improved by using tetrodes or other multi-electrode probes to record the activity of the same neurons from different nearby locations (Harris et al., 2000), enabling clustering to be performed in a space with dimensions defined both by waveform feature and by electrode identity. Here we analyzed tetrode recordings of neuronal activity using a Bayesian approach to spike-sorting (Sahani, 1999; Wehr et al., 1999) which performs automated clustering and provides principled estimates of the false-positive and false-negative rates for putative single-unit events. Unlike most other manual or automated spike-sorting algorithms, this spike-sorter corrects for potential peak misalignments due to undersampling, identifies and compensates for possible spike overlaps, and properly accounts for the statistical properties of background noise in the recordings. Nevertheless, there are many ways in which the characteristics of neural activity could still undermine the performance of this and other spike sorters.

Spike sorting can be undermined by distortions in spike waveform shape, for example arising from burst firing in individual neurons or overlap of coincident spikes from neighboring neurons. Changes in spike waveform shape with bursting can often be modeled successfully and incorporated into clustering algorithms (e.g., Sahani, 1999), but overlapping spikes are a much more difficult problem (Pillow et al., 2013; Rey et al., 2015). Waveform distortions from spike overlaps disrupt the clustering process if not identified and excluded before clustering, and even if successfully removed, overlaps reduce the number of waveform snippets providing useful information about distinctive single-unit waveform shapes. Here, we found that the distribution of threshold-crossing events suggested that burst firing was somewhat more common in recordings obtained under ket/med than urethane anesthesia (Figures 2A,B). However, the fraction of very short IEI in a recording was not predictive of spike-sorting success, indicating that neither increased burst firing nor increased rate of overlapping (near-coincident) spikes could explain poorer spike-sorting under ket/med than urethane anesthesia.

Spike sorting might also be undermined by coherent neuronal activity, even if action potentials from different neurons were not coincident enough to produce overlapping spikes. Coherent “population spiking”, in which local neuronal populations fire in a coordinated manner, is thought to be particularly prominent in the auditory cortex (Loebel et al., 2007). In principle, such coherent neuronal activity could complicate the initial filtering and thresholding stages of the spike-sorting process, by producing temporal correlations in background activity at long time-scales. Here, we found that extracellular voltages recorded on electrodes >150 μm apart were more coherent under ket/med than urethane anesthesia (Figures 4B,C), and multi-unit activity was more strongly correlated over time scales of tens of milliseconds (Figure 5). Our analysis also revealed that variance in raw voltage traces was elevated for ±100 ms around threshold-crossings in recordings made under ket/med anesthesia, but only ±3 ms under urethane anesthesia. We conclude that fluctuations in neuronal population activity over large spatial scales and long time scales undermine spike-sorting success under ket/med anesthesia.

### Implications for Understanding Auditory Cortical Activity Patterns

The most likely explanation for the findings reported here is that the majority of threshold crossings used for spike sorting do not originate from local spiking activity, but from high-frequency components of the raw extracellular voltage, riding on top of large-scale slow fluctuations in population activity that are much more pronounced under ket/med than urethane anesthesia. In the hippocampus, single-cell spike contributions to extracellularly recorded voltage signals can only be detected up to ~50 μm away from the cell (Henze et al., 2000). Large-amplitude deflections of the extracellular potential can arise from both spatial layout of the contributing sources and coordination of activity over time (Buzsáki et al., 2012). Most likely, the large-scale coordination of activity we observe is due to highly synchronized spiking activity across both cortical layers and frequency columns. The pattern of synchronization matches the spread of activity for both spontaneous and evoked activity observed in anesthetized and awake rat auditory cortex (Sakata and Harris, 2009): high coordination of activity within columns and coherence falling off more quickly with distance across columns. However, our data showed that the coordination of activity across columns falls off less steeply under ket/med than urethane anesthesia, pointing to difference in cortical activation patterns between these anesthetic regimes. Ketamine is known to induce a synchronized state in auditory cortex (Pachitariu et al., 2015), while under urethane, synchronized and de-synchronized states alternate (Luczak et al., 2007; Sakata and Harris, 2012; Pachitariu et al., 2015). The Timescale of these alternations is in the range of several 100 ms (Luczak et al., 2007), similar to the long-term slow fluctuations in population activity reported here (Figure 3).

It is possible that anesthesia-induced population synchronicity is particularly harmful to spike sorting for data obtained from primary auditory cortex. Induced activity in auditory cortex is highly transient (DeWeese et al., 2003) and strongly synchronized (DeWeese and Zador, 2006). Auditory cortex displays a specific distribution of cell-to-cell connections that has been shown to differ from other sensory cortices, including a higher portion of very strong connections that favor synchronized spiking (Atzori et al., 2001) and may underlie population spike dynamics specific to auditory cortex (Loebel et al., 2007).

### Possible Mechanisms Underlying Altered Population Activity Under Ket/Med

Ketamine blocks glutaminergic synapses (Franks, 2008), while urethane works at several sites, mostly enhancing GABAergic inhibition (Hara and Harris, 2002). Consequently, ketamine anesthesia appears to produce changes in temporal characteristics of responses in auditory cortex by reducing mostly late, un-synchronized responses (Wehr and Zador, 2005; Rennaker et al., 2007; Osanai and Tateno, 2016). This may lead to an overrepresentation of highly synchronized transient responses (DeWeese and Zador, 2006) in the recorded data. One explanation for the results reported here is thus a strong reduction of non-overlapping events in the late phases of neural responses recorded under ketamine anesthesia. Since the overall spread of activity throughout the cortex is very similar for evoked and spontaneous activity (Sakata and Harris, 2009), this alteration in the duration of evoked spiking might well extend also to bursts of spontaneous activity. In addition, it is possible that reduction of spiking in late phases of evoked activity (or spontaneous activity) under ketamine anesthesia might release from depression the particularly strong intracortical synapses found in auditory cortex (Atzori et al., 2001). Such release from depression could favor the spread of highly synchronized population spikes (Loebel et al., 2007).

### Consequences for Interpretation of Cortical Responses

It has been argued that functional cortical organization depends on anesthetic state (e.g., Guo et al., 2012). The results presented here provide an additional explanation for these results, since quality of spike-sorting may confound the measurement of single-cell response properties depending on brain state. The consequences of the observed dependency of spike-sorting success on brain state likely go beyond comparison of different anesthetic regimes. Spatial and temporal correlations of neural population activity in the auditory cortex reportedly depend not only on anesthetic state, but also on behavioral state (McGinley et al., 2015), age (de Villers-Sidani et al., 2010) and experience (Rothschild et al., 2013). Thus, apparent differences in the response properties of extracellularly recorded single units recorded under these different conditions might derive, at least in part, from the impact of cortical state on the spike-sorting process itself—or more specifically, on the initial thresholding step that is fundamental to every spike-sorting process.

## Author Contributions

KJH, MS and JFL conceived the study, interpreted the results and wrote the article. KJH collected and analyzed the data. JFL supervised experimental work.

## Conflict of Interest Statement

The authors declare that the research was conducted in the absence of any commercial or financial relationships that could be construed as a potential conflict of interest. The reviewer IC and handling Editor declared their shared affiliation.
